# AI-Powered Advances in Data Handling for Enhanced Food Analysis: From Chemometrics to Machine Learning

**DOI:** 10.3390/foods14193415

**Published:** 2025-10-03

**Authors:** Mourad Kharbach

**Affiliations:** Circular Economy/Sustainable Solutions, LAB University of Applied Sciences, Mukkulankatu 19, 15101 Lahti, Finland; mourad.kharbach@lab.fi

## 1. Introduction

The landscape of food analysis is being reshaped by the transformative power of data handling tools, including chemometrics, machine learning, and artificial intelligence (AI) [1]. These are not just an option; they are now an essential part of how we ensure food quality, safety, and authenticity [2]. Modern analytical instruments generate vast, complex datasets that are too large and intricate for traditional methods to handle [3]. From chromatography–mass spectrometry, which can detect hundreds of compounds in a single sample, to high-resolution imaging that captures minute textural details, the sheer volume of information has created an unprecedented need for advanced analytical power [4]. This Special Issue, “AI-Powered Advances in Data Handling for Enhanced Food Analysis: From Chemometrics to Machine Learning,” was conceived to explore and showcase this revolution, highlighting how the seamless integration of AI is reshaping our ability to ensure food quality, safety, and authenticity. It is a collective effort to share how researchers are moving beyond the limitations of classical statistical approaches and embracing the immense potential of machine learning and deep learning to unlock deeper insights from food data.

## 2. Bridging the Knowledge Gap: From Chemometrics to AI

For decades, chemometrics has been the workhorse of food analysis. Techniques like Principal Component Analysis (PCA) [5] and Partial Least Squares Regression (PLSR) [6] have been instrumental in extracting information from multivariate data. However, the sheer volume and dimensionality of data from high-throughput technologies like chromatography–mass spectrometry and spectroscopy often overwhelm these classic methods [7]. A significant knowledge gap emerged between the capabilities of conventional chemometrics and the demands of modern data.

This is where machine learning and AI have stepped in, filling a crucial void. Algorithms such as Support Vector Machines [8], Random Forests [9], and Artificial Neural Networks [10] are adept at handling large, high-dimensional datasets and uncovering complex, non-linear relationships that traditional methods often miss. As demonstrated by the recent literature, the fusion of spectroscopic data with machine learning has enabled rapid, non-destructive quality control for a wide range of food products—from meat to dairy [1]. Similarly, deep learning has proven effective for fine-grained visual classification of foods, a task previously challenging due to high inter-class similarity [11]. This Special Issue sought to bridge this gap, not by replacing chemometrics, but by showing how AI can amplify its capabilities and enable [11] more accurate and robust analytical models. We featured research that demonstrated the tangible benefits of integrating these approaches, from improving classification accuracy in food authentication to streamlining complex quality control processes.

## 3. Future Research Directions: Unlocking New Potential

The advancements presented in this Special Issue provide a roadmap for the future of food analysis, but several key areas still need to be addressed to truly unlock AI’s full potential.

While AI has already revolutionized food analysis, several key areas must be addressed to unlock its full potential. The first is the critical need for explainable AI (XAI) [12]. Many powerful deep learning models operate as “black boxes,” making it difficult for researchers and regulatory bodies to understand how a specific decision was reached. Future research should focus on developing models that are not only accurate but also interpretable, providing clear insights into the underlying chemical and physical properties that drive a prediction.

Another promising avenue is multi-omics integration. By using AI to fuse data from genomics, metabolomics, and proteomics with conventional analytical data, we can create a more holistic and comprehensive understanding of food products [13]. This approach holds the potential to solve complex challenges, from identifying subtle markers of food fraud to understanding the full impact of food processing on nutritional content.

Finally, the field needs standardization and validation frameworks for AI-based methods. For these powerful tools to be widely adopted in industry and accepted by regulatory agencies, there must be a consensus on best practices, data sharing protocols, and model validation procedures. By focusing on these areas, we can ensure that AI-driven food analysis becomes a reliable and trusted cornerstone of a safer, higher-quality, and more transparent global food system.

## 4. Conclusions

This Special Issue has successfully brought together a collection of cutting-edge research that underscores the pivotal role of AI in advancing food analysis. By showcasing the latest developments and applications, from the enhancement of classic chemometric techniques to the innovative use of deep learning, we have demonstrated how AI-powered data handling tools are providing unprecedented insights into food safety, quality, and authenticity. We hope this collection will serve as a valuable resource and inspire a new wave of research and collaboration. The journey toward a fully data-driven food science is well underway, and with continued innovation, we can harness the full power of AI to build a more secure and trustworthy global food system.

## 5. An Overview of Published Articles

Twenty-three manuscripts were submitted for the Special Issue, and all of them were subject to the rigorous Foods review process. In total, seven papers were accepted for publication in this Special Issue (six articles and one review). The contributions are briefed below:

Jule Hansen et al., 2025 (contribution 1) perfectly illustrates the Special Issue’s focus on food authenticity and fraud detection. The work by Jule Hansen et al. tackles the critical need for verifying apple provenance, variety, and cultivation methods. It exemplifies the integration of a powerful analytical technique, UHPLC-Q-ToF-MS, with a robust machine learning algorithm, Random Forest. The authors address a key challenge the complexity of analyzing a single product for multiple authentication questions by demonstrating that one analytical approach can yield multiple classification models. This highlights a crucial theme: leveraging AI to maximize the value of analytical data and streamline complex analyses. The variable selection aspect also touches upon the future trend of model explainability, showing which parts of the data are most important for different classification tasks, which helps to build trust in the models.

Zhiyu Zhao et al. (contribution 2) is a prime example of the Special Issue’s forward-looking approach; specifically, its emphasis on XAI. The study uses Random Forest Regression not just for prediction, but to understand the complex relationships between phenolic compounds, amino acids, and antioxidant activities in fermented apricot kernels [5]. This work goes beyond simply demonstrating high predictive accuracy and directly addresses a major knowledge gap: the “black box” nature of many AI models. By identifying specific amino acids and phenolic compounds that positively impact antioxidant activity, the authors provide clear, actionable insights. This bridges the gap between AI-driven prediction and a fundamental scientific understanding of food chemistry, paving the way for future research that is both data-driven and interpretable.

Zengzheng Chen et al.’s (contribution 3) work on food image recognition delves into the cutting-edge application of deep learning for food analysis. It addresses the challenges of Fine-Grained Visual Classification, where subtle differences between food images can be difficult for models to distinguish. The proposed Multi-level Attention Feature Fusion Network and a novel sub-class center loss directly tackle the knowledge gap of effectively capturing intricate local features while minimizing intra-class variability. This research moves beyond standard deep learning applications, pushing the boundaries of what is possible with computer vision in food science and demonstrating the future of AI in fields like dietary monitoring and quality control.

Wenwen Zhang et al. (contribution 4) highlights a key goal of the Special Issue: the application of AI for rapid and non-destructive quality control in the food industry. The authors compare different predictive models, including XGBoost, CNN, and ResNet, to determine moisture content in *Porphyra yezoensis* using near-infrared spectroscopy. The study provides a clear example of model comparison and optimization, recommending the most reliable and accurate model (XGBoost) for industrial application. Furthermore, the use of Gaussian Process Regression for uncertainty assessment reinforces the critical future trend of ensuring model reliability, which is essential for the widespread adoption of AI in real-world food production environments.

Haijun Du et al.’s work (contribution 5) on determining the crude protein content of alfalfa is a perfect illustration of how chemometrics and AI can be effectively integrated. The study showcases a hybrid approach using Fourier Transform Infrared Spectroscopy (FTIS) alongside a combination of data preprocessing techniques (airPLS, Savitzky–Golay) and feature selection methods (CARS) before applying both classical chemometric models (PLSR) and machine learning algorithms (Random Forest Regression). The high predictive performance achieved, particularly with the combined PLSR model, demonstrates that robust, small-sample prediction models can be built by leveraging the strengths of both traditional and modern data handling tools, directly addressing a key theme of this Special Issue.

Yu Song et al. (contribution 6) tackles a fundamental challenge in food chemistry: the time-consuming process of taste determination. Yu Song et al.’s research brilliantly demonstrates how computational techniques and machine learning can expedite this process. The use of Graph Neural Networks (GNNs) to model molecular structures is a cutting-edge approach that goes beyond traditional molecular descriptors. By showing that GNNs and “consensus models” that combine multiple representations outperform other methods, the authors highlight a significant future trend: the use of advanced deep learning models to understand complex relationships between molecular structure and sensory properties. This work opens up new avenues for food design and innovation.

Achilleas Karamoutsios et al. (contribution 7) provides a comprehensive overview of how proteomics and chemometrics are being combined to combat economically motivated food adulteration, specifically in milk. The authors highlight the transition from traditional methods to modern, high-resolution techniques and their subsequent integration with chemometric approaches like PCA and PLS-DA. The review article successfully identifies the key knowledge gap in milk authenticity—the need for standardization, broader validation, and data fusion. By advocating for the future convergence of proteomics with multi-omics integration and machine learning frameworks, this work provides a roadmap for the future research highlighted in the Special Issue, pushing the field towards more scalable, specific, and robust solutions for complex food systems.
